# Multiple-Coated PLGA Nanoparticles Loading Triptolide Attenuate Injury of a Cellular Model of Alzheimer's Disease

**DOI:** 10.1155/2021/8825640

**Published:** 2021-02-25

**Authors:** Lu Jia, Xiao-qi Nie, Hong-ming Ji, Zhi-xiang Yuan, Rong-shan Li

**Affiliations:** ^1^Second Hospital of Shanxi Medical University, Taiyuan, Shanxi, China; ^2^Shanxi Provincial People's Hospital, Taiyuan, Shanxi, China; ^3^College of Pharmacy, Southwest Minzu University, Chengdu, Sichuan, China

## Abstract

Alzheimer's disease (AD) is the most common neurodegenerative disease, which is associated with extracellular deposition of amyloid-*β* proteins (A*β*). It has been reported that triptolide (TP), an immunosuppressive and anti-inflammatory agent extracted from a Chinese herb *Tripterygium wilfordii*, shows potential neuroprotective effects pertinent to AD. However, the clinical use of TP for AD could be hampered due to its high toxicity, instability, poor water solubility, and nonspecific biodistribution after administration. In this paper, we reported a kind of multiple-coated PLGA nanoparticle with the entrapment of TP and surface coated by chitosan hydrochloride, Tween-80, PEG20000, and borneol/mentholum eutectic mixture (MC-PLGA-TP-NP) as a novel nasal brain targeting preparation for the first time. The obtained MC-PLGA-TP-NP was 147.5 ± 20.7 nm with PDI of 0.263 ± 0.075, zeta potential of 14.62 ± 2.47 mV, and the entrapment efficiency and loading efficiency of 93.14% ± 4.75% and 1.17 ± 0.08%, respectively. In comparison of TP, MC-PLGA-TP-NP showed sustained-release profile and better transcellular permeability to Caco-2 cells in vitro. In addition, our data showed that MC-PLGA-TP-NP remarkably reduced the cytotoxicity, attenuated the oxidative stress, and inhibited the increase of the intracellular Ca^2+^ influx in differentiated PC12 cells induced by A*β*_1-42_. Therefore, it can be concluded that MC-PLGA-TP-NP is a promising preparation of TP, which exerts a better neuroprotective activity in the AD cellular model.

## 1. Introduction

Alzheimer's disease (AD), the most common neurodegenerative disease, is a major form of dementia characterized clinically by progressive cognitive impairments, including memory, judgment, decision-making, and language [1, 2]. However, the pathogenesis and etiology of AD are still far from well understanding. The pathological changes observed in brains of AD patients reflect a series of chronic inflammatory processes, in which response is characterized by the presence of abundant activated microglia. Moreover, mounting evidence indicates that reactive astrocyte proliferation is associated with extracellular deposition of amyloid-*β* proteins (A*β*), formation of neurofibrillary tangles and amyloid plaques, and increased expression of proinflammatory cytokines and complement components, which in turn induce neuroinflammation in the brain [3, 4]. Therefore, more and more studies have focused on the roles and mechanisms of anti-inflammatory and immune modulatory agents for AD treatment.

Triptolide (TP), a major bioactive ingredient extracted from the roots, leaves, flowers, and fruits of *Tripterygium wilfordii* widely used as a herbal medicine [5], has been shown to possess multiple pharmacological functions [6, 7], including potential neuroprotective effects pertinent to AD [8]. However, the clinical use of TP is hampered due to its high toxicity, instability, poor water solubility, and nonspecific biodistribution after administration. Additionally, the major hurdle for delivering drugs to brain is the presence of the blood-brain barrier (BBB) which is the complicated network of blood vessels having tightly packed endothelial cells for the separation of the brain from the blood circulatory system [9]. Thus, in order to realize the efficient therapy of TP for AD, it is necessary to develop novel TP preparation for overcoming the mentioned limitation.

With the rapid development of nanotechnology, biodegradable polymers have been extensively applied in pharmaceutical and biomedical formulations during the past few decades [10]. Especially, poly(D,L-lactic-co-glycolic acid) (PLGA) approved by the Food and Drug Administration (FDA) has been successfully applied for the formation of nanoparticles in lab and in clinic because of good biocompatibility and biodegradability [11]. The entrapment of TP into PLGA nanoparticles is considered a smart way to increase the stability and water solubility, decrease the toxicity, and enhance the specific distribution of TP after administration [12]. Intranasal administration could noninvasively realize drug-loaded nanoparticles to bypass the BBB to deliver therapy agents for the treatment of brain disease by the olfactory pathway which is a reliable alternative to achieve desire therapeutic effects at lower doses while minimizing side effects [9, 13]. Therefore, the development of TP-loaded PLGA nanoparticles is an ideal method to improve the treatment for AD.

In previous study, we have developed a novel delivery system to promote targeting profile and reduce toxicity of TP [14]. In our current work, we focus on bypassing the BBB using intranasal administration of PLGA nanoparticles coated by chitosan hydrochloride, Tween-80, PEG20000, and borneol/mentholum eutectic mixture. TP was also encapsulated into the multiple-coated PLGA nanoparticles as a novel brain targeting preparation (MC-PLGA-TP-NP) for nasal drug delivery with the aim of overcoming the clinical limitation. Then, MC-PLGA-TP-NP was characterized, and its cumulative release was evaluated. To prove the better permeation to the olfactory neurons, we performed the transcellular permeability study on MC-PLGA-TP-NP through Caco-2 monolayer cells. In addition, a cellular model of AD (the differentiated PC12 cells) induced by A*β*_1-42_ was established to evaluate the attenuation of cytotoxicity, reactive oxygen species (ROS) generation, and calcium influx after the treatment of MC-PLGA-TP-NP using free TP as a contrast.

## 2. Materials and Methods

### 2.1. Chemicals and Reagents

Triptolide (TP, purity >99%), PLGA (with a copolymer ratio of lactide : glycolide 50 : 50 and Mw : 40-75 kDa), poloxamer 188 (F-68, Mw : 8350 Da), amyloid beta-peptide_1-42_ (A*β*_1-42_), and 3-(4,5-dimethylthiazol-2-yl)-2,5-diphenyltetrazolium bromide (MTT) were supplied by Sigma-Aldrich Co. Ltd. (Gillingham, UK). Dichlorodihydrofluorescein diacetate (DCFH-DA), fluo-3 acetoxymethyl ester (fluo-3-AM), LysoTracker Red, and 4′,6-diamidino-2-phenylindole (DAPI) were from Molecular Probes (Eugene, OR, USA). Fetal bovine serum (FBS), horse serum (HS), nerve growth factor (NGF), penicillin, and streptomycin were provided by GIBCO, Invitrogen Corporation (Carlsbad, CA, USA). Chitosan hydrochloride (degree of deacetylation, 80% ~90%, viscosity, 30 mPa.s) was obtained from Shanghai KABO Trading Company (Shanghai, China). Polyethylene Glycol (PEG, MW = 20 kDa), borneol, mentholum, and Tween-80 were purchased from Chengdu Kelong Chemical Company (Chengdu, China). The other chemicals were of analytical reagent grade.

### 2.2. Preparation of MC-PLGA-TP-NP

MC-PLGA-TP-NP was prepared according to our previous method with minor modification [13]. In brief, 415 mg PLGA, 5 mg TP, and 150 mg borneol/mentholum eutectic mixture (1 : 3, w/w) were dissolved into 50 ml of ethanol/acetone (3 : 2) followed by a dropwise addition to 50 ml of 1% w/v poloxamer 188 solution containing 250 mg PEG with continuous stirring on a magnetic stirrer at room temperature to obtain a dispersion. After vacuum rotary evaporation to remove the organic solvent, 250 mg Tween-80 and 50 mg chitosan hydrochloride were added to the dispersion while being stirred magnetically for 2 h. Then, the dispersion was centrifuged at 25 000 rpm for 30 min. The supernatant was collected for HPLC analysis while the precipitate was obtained, namely, MC-PLGA-TP-NP, which was dispersed into 10 ml ultrapure water containing 0.5 g of lactose as lyoprotectant. The obtained dispersion was prefrozen using ultra-low temperature refrigerator at -80°C for 24 h followed by lyophilization in freeze-dryer equipment (LyoQuest, Telstar, Spain) under 0.4 mbar vacuums for 24 h and a condenser temperature of -40°C to form a powder for storage at 4°C.

### 2.3. Entrapment Efficiency and Loading Efficiency

HPLC (Aglient 1260, Aglient Technologies, Santa Clara, CA) was equipped with an online degasser, a quaternary pump, an autosampler, a column thermostat, and an Aglient C18 (250 mm × 4.6 mm, 5 *μ*m) analytical column for chromatographic separation. All separations were executed at 30°C with a mobile phase of acetonitrile/water (25 : 75), a flow rate of 1.0 ml/min, and a 10 *μ*L sample volume injected into the HPLC system for analysis. The UV detector was operated at 218 nm [14, 15]. Peak identification was carried out by comparing the retention times of the samples with those of reference standards. The centrifugation method was involved to calculate the entrapment efficiency and loading efficiency. The collected supernatant from the dispersion after the centrifugation at 25 000 rpm was diluted appropriately and then injected into HPLC to assess the amount of TP in the supernatant. Entrapment efficiency (EE) and loading efficiency (LE) were determined according to the following equation [16].

EE% = (total amount of TP − the amount of TP in the supernatant)/total amount of TP × 100%.

LE% = (total amount of TP − the amount of TP in the supernatant)/[(total amount of TP − the amount of TP in the supernatant) + total amount of PLGA] × 100%.

### 2.4. Characterization of MC-PLGA-TP-NP

MC-PLGA-TP-NP freeze-dried powder was appropriately dispersed with distilled water. The particle size and zeta potential of MC-PLGA-TP-NP were determined by Malvern Zetasizer Nano ZS90 (Malvern Instruments Ltd, Malver, UK). S4800 Transmission electron microscope (Hitachi Ltd, Tokyo, Japan) was applied to observe the features of MC-PLGA-TP-NP. Briefly, MC-PLGA-TP-NP suspension was dripped onto a dedicated copper mesh and then negatively stained with 4% phosphotungstic acid solution for 30 s. After drying at ambient temperature, the nanoparticles were observed under the transmission electron microscope.

### 2.5. In Vitro Release

In vitro release studies were carried out in 0.05 M phosphate buffer solutions (PBS, containing 0.1% Tween 80) at pH 4.0 and pH 7.4 using dynamic membrane dialysis. 5 ml of MC-PLGA-TP-NP or TP suspension (with an equivalent dose of 5 mg TP) was added in a dialysis bag (3500 Da) and then dialyzed against 95 ml PBS in 250 ml beaker situated in oscillating water bath at a temperature of 37 ± 0.5°C. Samples (1 ml) were obtained from the release medium at arranged time points (5, 30 min, 1, 2, 4, 8, 12, and 24 h) for determining the amount of TP that diffused through the dialysis bag. Meanwhile, 1 ml of fresh release medium preheated at 37°C was added to maintain a constant volume at the above prearranged time points. Samples were analyzed by the HPLC method as described previously. Cumulative release characteristics of MC-PLGA-TP-NP or TP suspension were described and compared.

### 2.6. Cell Culture

The human epithelial cell line (Caco-2) purchased from American Type Culture Collection (Rockville, Maryland) was cultured in DMEM medium containing 10% FBS, 2 mM glutamine, 100 *μ*g/ml streptomycin, and 100 U/ml penicillin and maintained in a humidified atmosphere containing 5% CO_2_ at 37°C. Rat pheochromocytoma (PC12) cells kindly provided by Chinese Academy of Sciences were grown in RPMI1640 supplemented with 5% FBS, 10% HS, 100 *μ*g/ml streptomycin, and 100 U/ml penicillin and incubated in a humidified atmosphere containing 5% CO_2_ at 37°C. Then, PC12 cells were plated onto 6-well culture plates or 96-well culture plates coated with poly-D-lysine at a density of 1 × 10^5^, and the medium containing 2.5 ng/ml NGF was replaced 2~3 times for a week to obtain neuronal differentiated PC12 cells. All subsequent experiments in the present study were performed with the differentiated PC12 cells.

### 2.7. Transcellular Permeability

Caco-2 cells were seeded on the apical chamber of 6-well Transwell™ plates (Corning Inc., Corning, New York) at a density of 1.0 × 10^5^ cells/cm^2^. The DMEM-based culture medium was replaced with fresh medium every two days for about three weeks until they achieved a constant transepithelial electrical resistance (TEER, >500 *Ω*·cm^2^), indicating the formation of tight junctions in the monolayer. The apical (top) and basal (bottom) chambers were separated by a collagen-coated polytetrafluoroethylene membrane (4.52 scm^2^ surface, 24 mm diameter) with a pore size of 0.4 *μ*m. Before the initiation of the experiments, the culture medium was removed, and the cell membranes were allowed to equilibrate in Hank's Balanced Salt Solution (HBBS, transport buffer) as follows: HBSS buffered with 10 mM MES (pH 6.0) or 10 mM HEPES (pH 7.4) was added to the apical (1.5 ml) or basal chamber (2.5 ml), respectively. After preincubation with transport buffer for 30 min, permeability experiments were initiated by the addition of TP solution or MC-PLGA-TP-NP to the apical chamber at a final TP concentration of 20 *μ*g/well in HBBS. At prearranged time intervals (15, 30, 60, and 120 min), an aliquot of 200 *μ*l sample was collected from the basal chamber and replaced by 200 *μ*l of a prewarmed HBSS buffer to maintain the total volume. Samples were analyzed by the HPLC method as described previously. Each experiment was repeated five times (*n* = 5). The cumulative permeability curves versus time of TP were plotted, and the apparent permeability coefficients (*P*_app_) were calculated using the equation as follows [17]:

(1) $$\begin{array}{c} P_{app}=\left(\Delta Q/\Delta t\right)\times \left[1/\left(A\times C_{0}\right)\right]. \end{array}$$

*ΔQ*/*Δt* (*μ*mol/s) is the rate at which TP appears in the basal chamber, *C*_0_ (*μ*mol/L) means the initial concentration of TP in the apical chamber, and *A* (cm^2^) represents the surface area of the cell monolayer.

### 2.8. Effects of TP or MC-PLGA-TP-NP on A*β*_1-42_-Induced Cytotoxicity in PC12 Cells

According to previous study from Wang et al., 5 *μ*M A*β*_1-42_ as a final concentration was incubated with the differentiated PC12 cells to induce the cytotoxicity for evaluating the protective effects of TP and MC-PLGA-TP-NP [18]. Different concentrations of TP solution (0 ng/ml, 0.1 ng/ml, 0.5 ng/ml, 1.0 ng/ml, 5.0 ng/ml, and 10 ng/ml) and MC-PLGA-TP-NP (with an equivalent dose of TP solution) were added to the differentiated PC12 cells, respectively. The medium containing 1% DMSO was served as control. After the incubation for 24 h, the cell viability was determined by the MTT assay. Each experiment was repeated five times (*n* = 5).

### 2.9. Measurement of ROS Generation

The production of intracellular ROS induced by A*β*_1-42_ under different conditions was measured by dichlorodihydrofluorescein diacetate (DCFH-DA), a membrane-permeable probe. The differentiated PC12 cells were exposed to 5 *μ*M A*β*_1-42_, and then TP solution or MC-PLGA-TP-NP (with an equivalent dose of TP solution) was added to provide 5.0 ng/ml of TP concentration for 24 h, respectively. The cells exposed to the medium only were served as control. After incubation with 10 *μ*M DCFH-DA at 37°C for 1 h, cells were washed with PBS for three times. The methodology was performed according to the procedures described in the ROS assay kit, and all procedures were carried out in the dark. The intracellular accumulation of ROS was immediately analyzed by a CytomicsFC 500 flow cytometry (Beckman Coulter, USA). 10,000 events were collected, and the level of ROS generation was calculated as follows: the level of ROS (%) = the percentage of DCF − positive cells in the M1 region [19]. Each experiment was repeated five times (*n* = 5).

### 2.10. Measurement of Calcium Influx

Changes in intracellular calcium concentrations were monitored using Fluo-3-AM, a probe for Ca^2+^ measurement. Similarly, the differentiated cells seeded in 6-well plate were exposed to 5 *μ*M A*β*_1-42_, and then TP solution or MC-PLGA-TP-NP was added to provide 5.0 ng/ml of TP concentration, respectively. The cells exposed to the medium only were served as control. After incubation for 48 h, 3 *μ*M Fluo-3-AM dissolved in HEPES solution (5.5 mM HEPES, 140 mM NaCl, 5 mM KCl, 1 mM CaCl_2_, 0.5 mM MgCl_2_, 10 mM glucose, 1% DMSO, pH 7.4) was added into the chamber for another incubation at 37°C for 40 min. Then, the cellular lysosomes and nucleus were stained with LysoTracker Red (50 nM) and DAPI (1 mg/ml) at room temperature for 30 min, respectively. Finally, the cells were rinsed with HEPES solution for three times prior to the observation under Axio observer D1 fluorescence microscope (Carl Zeiss, Germany) with the excitation at 405 nm and 488 nm using a green channel (523 nm barrier filter), a red channel (630 nm barrier filter), and a blue channel (480 nm barrier filter). The measurement of fluorescence data (Fluo-3-AM) from individual cells was carried out using the MetaMorph software (Version 7.1, Universal imaging, USA).

### 2.11. Data Analysis

Data were compared with the SPSS 15.0 statistical package. Multiple comparisons of mean values were performed by one-way ANOVA with Fisher's least significant difference (LSD) test applied for posthoc comparisons at 95% confidence interval. *p* < 0.05 was considered statistically significant.

## 3. Results and Discussion

It is reported that TP as a herb-derived drug shows a broad spectrum of biological profiles including anti-inflammatory, immunosuppressive, antifertility, antitumor activities, and neurotrophic and neuroprotective effects [7]. Nevertheless, the clinical use of TP is often discouraged due to its high toxicity, poor water solubility, and nonspecific biodistribution. Thus, how to enhance its solubility and improve targeting profile are key issues to be solved by developing new preparations. Various nanoparticles as useful vehicles were widely designed to load TP for the improvement of its shortcomings, including polylactic acid nanoparticle [20], solid lipid nanoparticle [21], and lipid-polymer hybrid nanoparticle [22]. In the study, a kind of multiple-coated PLGA nanoparticle by chitosan hydrochloride, Tween-80, PEG20000, and borneol/mentholum eutectic mixture was fabricated to load TP with the aims of enhancing the permeability of TP through BBB and exerting strong neuroprotective effects for the first time. As described in our previous study, free drugs can directly through the olfactory region to reach the cerebrospinal fluid or even the brain after intranasal administration. However, the most amount of drug will be suffered from adsorption and degradation under the action of the nasal enzymes and nasal mucosa, causing a very low amount of drug in the brain [13]. In order to increase the concentration of TP in the brain by nose-to-brain delivery, PLGA nanoparticles were used to protect TP from degradation and sustain release of TP while the coating of Tween-80 and PEG on the surface of nanoparticles could avoid the clearance by macrophage and inhibit P-glycoprotein in the brain capillary endothelial cell membrane for the drug efflux function. Besides, the addition of chitosan hydrochloride and borneol/mentholum eutectic mixture is an effective way to modulate the permeability of BBB to facilitate the entry of therapeutic drugs into the brain.

HPLC analysis results showed that the retention time of TP was approximately 17.0 min. The calibration curves of TP (*A* = 37857C + 13127, *R*^2^ = 0.9997. *A* and *C* represented peak area and concentration, respectively), linear over the range of 2.16-54.0 *μ*g/ml, were used to calculate the concentration of TP in the supernatant from the dispersion. The methodology recoveries were among 95~103% for all the three concentrations (10, 20, 40 *μ*g/ml). Additionally, intraday and interday precision of TP was determined to be 1.74% and 2.64%, respectively. Using the EE and LE as indexes determined by HPLC, the preparation process and formulation screening were performed according to our preliminary experiments (data not shown). After lyophilization in freeze dryer for 24 h, the optimal MC-PLGA-TP-NP was obtained with the average EE of 93.14% ± 4.75% as well as the average LE of 1.17 ± 0.08%.

**Figure 1:**
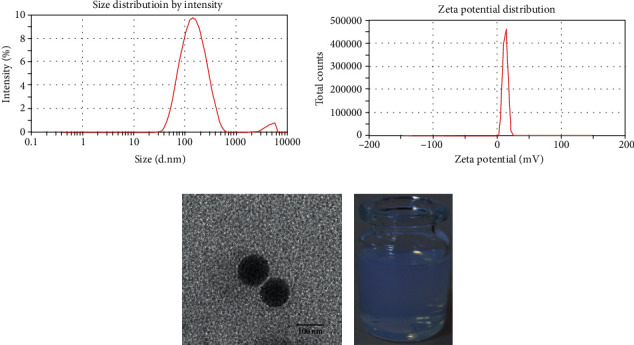
Size distribution (a), zeta potential distribution (b), and TEM image (c) of MC-PLGA-TP-NP redispered into deionized water (d).

The obtained MC-PLGA-TP-NP was well redispersed into deionized water (a sky-blue colored colloidal solution, Figure 1(d)) for the measurement of the particle size and zeta potential. As shown in Figures 1(a) and 1(b), the average particle size of the MC-PLGA-TP-NP was 147.5 ± 20.7 nm with PDI of 0.263 ± 0.075 and zeta potential of 14.62 ± 2.47 mV. As indicated in Figure 1(c), MC-PLGA-TP-NP was approximately spherical with a certain uniform size distribution. The surface morphology was found to be relatively smooth, and no surface adsorbed particles were observed. There was a small peak appeared at about 4 *μ*m in the particle-size distribution graph (Figure 1(a)), which might be caused by the redispersion after lyophilization.

**Figure 2:**
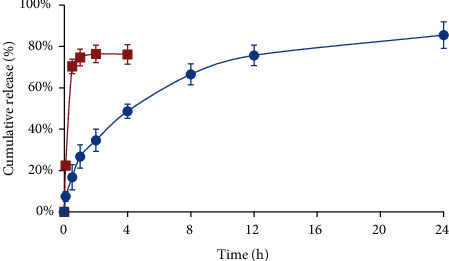
In vitro release profile of TP suspension (■) or MC-PLGA-TP-NP (●) in PBS (pH = 7.4) using the dialysis bag diffusion technique. The experiment was repeated in triplicate, and data were given as the mean ± standard deviation (*n* = 3).

In vitro release behavior of TP suspension or MC-PLGA-TP-NP in PBS (pH = 7.4) was evaluated by dynamic membrane dialysis [23]. As shown in Figure 2, a rapid release within 0.5 h (about 70%) was observed for TP suspension and then reached a plateau within 2 h (about 80%). The release rate of TP from MC-PLGA-TP-NP was also evaluated. The first phase was a 26.75% release within the first 1 h mainly because TP adsorbed on the surface of nanoparticles could quickly diffuse in the PBS initially. And then a slow release, the second phase, was afterward for a period of 24 h, which may be caused by TP diffusion from PLGA matrix. The maximum release rate of TP from MC-PLGA-TP-NP was more than 80% within 24 h, indicating sustained-release profile of nanoparticles, which might be helpful for promoting the efficacy of neurotherapeutics. Notably, the cumulative release rate for both TP suspension and MC-PLGA-TP-NP in PBS were much less than 100%, maybe because of the degradation of TP in the release medium.

**Figure 3:**
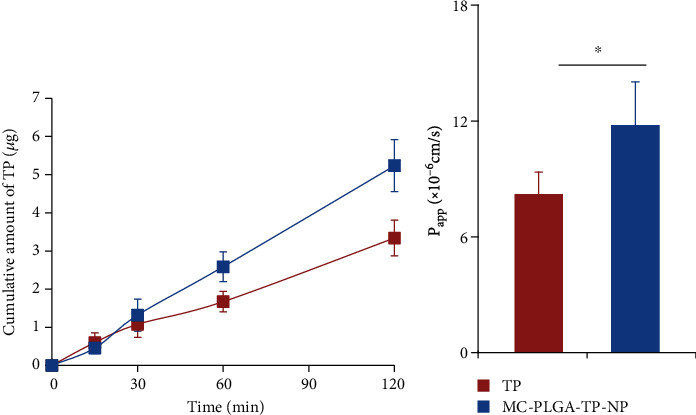
Transcellular permeability study on MC-PLGA-TP-NP through Caco-2 cells. (a) The time profiles of TP or MC-PLGA-TP-NP permeation through the Caco-2 monolayer seeded on Transwell plates; (b) The comparison of the calculated *P*_app_ of TP and MC-PLGA-TP-NP. The experiment was repeated five times, and data were represented as the mean ± standard deviation (*n* = 5). ^∗^Significant difference (*p* < 0.05, versus TP).

Deep administration of drugs to the nasal cavity approached nasal mucosa, leading to direct transmission of drug into the brain via the olfactory pathway, which consists of olfactory neurons with ability to carry drugs from olfactory mucosa to the brain [9]. As reported, nanoparticles, as excellent platforms for direct delivery of drug to the brain after intranasal administration by protecting drug from biological and chemical degradation and inhibiting the efflux of P-glycoprotein, were designed to encapsulate and deliver various therapeutic agents across the olfactory pathway to the brain [24–26]. It is unknown if the designed MC-PLGA-TP-NP was involved in the intracellular transport. We assumed that it could be based on literature mentioned above. Herein, we performed the transcellular permeability study on MC-PLGA-TP-NP through Caco-2 cells. Figures 3(a) and 3(b) shows the time profiles and the calculated *P*_app_ of TP or MC-PLGA-TP-NP permeation through the Caco-2 monolayer seeded on Transwell plates. The results demonstrated that free TP molecules exhibited better transcellular permeability than MC-PLGA-TP-NP at the beginning, whereas the amount of MC-PLGA-TP-NP increased sharply after incubation for 30 min. The level of determined TP in the basal chamber of the MC-PLGA-TP-NP group was 1.5-fold higher than that of the TP group at 120 min (Figure 3(a)). The *P*_app_ of free TP calculated using the equation of Artursson and Karlsson was 8.219 ± 1.145 × 10^−6^ cm/s, which was consistent with the data from Gong et al. [17]. Obviously, The *P*_app_ of MC-PLGA-TP-NP was much higher (11.79 ± 2.242 × 10^−6^ cm/s, Figure 3(b)), indicating that the designed nanoparticles could enhance the transcellular permeability of TP. According to the design of Transwell™ permeable supports, it is expected that drug permeated by passive absorption through transcellular or paracellular mechanisms can be observed in the basolateral compartment [27]. In this study, it is believed that the amount of TP determined in the basal chamber for both free TP and MC-PLGA-TP-NP can be attributed to the combination of passive transcellular and paracellular absorption mechanisms. In contrast, the higher TP amount in the basal chamber from MC-PLGA-TP-NP indicated that the multiple chemical coatings on the surface of nanoparticles can open intercellular junctions to accelerate the transcellular permeability of TP.

**Figure 4:**
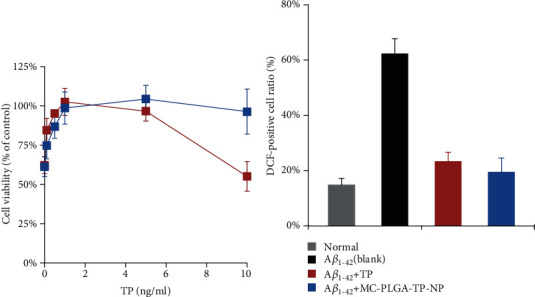
The attenuation of cytotoxicity (a) and ROS generation (b) in the differentiated PC12 cells induced by A*β*_1-42_ after the incubation with TP or MC-PLGA-TP-NP for 24 h. The experiment was repeated five times, and data were represented as the mean ± standard deviation (*n* = 5).

A*β* peptides, predominantly A*β*_1-40_ and A*β*_1-42_, are derived from the separation of the amyloid precursor protein [28]. Plenty of evidence have shown that an abnormal deposition of A*β* peptides in plaques of the brain tissue could cause the loss of neurons and synaptic lesions, which was considered the primary cause of the pathogenesis of AD [29]. At least partially, A*β* neurotoxicity is induced by oxidative stress. Hence, therapeutic effort for attenuating the oxidative stress could be beneficial to AD treatment [19]. In the present study, we investigated the functions of TP and MC-PLGA-TP-NP in the cytotoxicity of A*β*_1-42_ on oxidative stress. Wang et al. reported that A*β*_1-42_ was cytotoxic to differentiated PC12 cells in a concentration-dependent manner. Herein, 5 *μ*M A*β*_1-42_ as a final concentration was selected to induce the cytotoxicity for evaluating the protective effects of TP and MC-PLGA-TP-NP. As shown in Figure 4(a), A*β*_1-42_ obviously produced cytotoxicity without the protection of TP or MC-PLGA-TP-NP (0 ng/ml points), and only about 60% differentiated PC12 cells survived after incubation for 24 h. With the increase of TP dosage in the TP group, the viability of PC12 cells increased until the concentration of 5 ng/ml of TP reached, which was consistent with the results from Xu's report [19]. However, the viability of PC12 cells suffered from a rapid decrease to about 50% when the concentration of TP was raised from 5 ng/ml to 10 ng/ml, indicating that high dosage of TP could cause stronger toxicity and even reverse the protection effect. Notably, no obvious cytotoxicity was observed after the PC12 cells incubated with MC-PLGA-TP-NP in the whole range of prearranged concentrations. The reason may be that the sustained-release profile of MC-PLGA-TP-NP avoided the high dosage of TP interacting with cells. The results were acceptable and understandable because TP was a toxic chemical according to our previous study [6]. Thus, the development of new TP preparation to reduce its toxicity is a necessity.

It was reported that the protective functions of TP in AD may be via the pathway of oxidative stress. To evaluate the attenuation of the oxidative stress, the measurement of ROS generation closely associated with oxidative stress was carried out in this study. The results showed that the contents of ROS in the differentiated PC12 cells were increased significantly after the incubation with A*β*_1-42_ (*p* < 0.05). Fortunately, the levels of intracellular ROS in differentiated PC12 cells for the treatment of both TP and MC-PLGA-TP-NP were significantly reduced compared to the blank group which was treated with A*β*_1-42_ only (Figure 4(b)), respectively, indicating that the treatment of the differentiated cells with TP or MC-PLGA-TP-NP can attenuate oxidative stress induced by A*β*_1-42_. There was no significant difference between the TP and MC-PLGA-TP-NP group (*p* > 0.05). Taken together, the designed MC-PLGA-TP-NP can exert an attenuation of the oxidative stress without any cytotoxicity to the differentiated PC12 cells.

**Figure 5:**
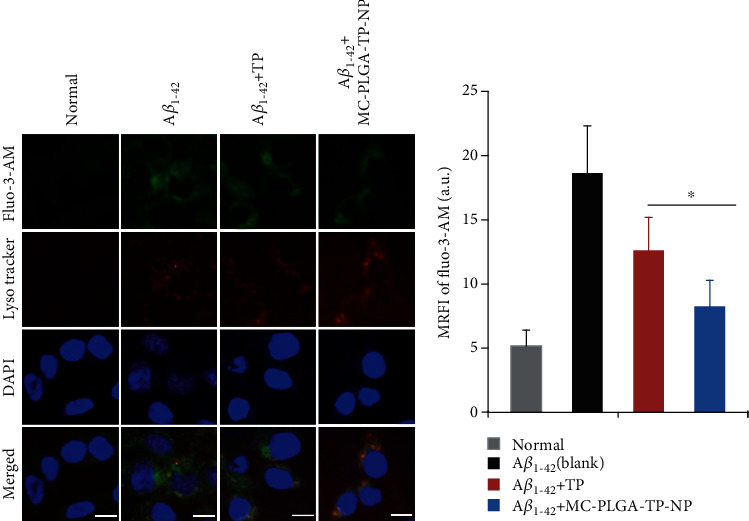
Fluorescence observation of calcium influx in the differentiated PC12 cells stained by different molecular probes. (a) The fluorescence images of the differentiated PC12 cells induced by A*β*_1-42_ after the incubation with TP or MC-PLGA-TP-NP for 48 h. (b) Statistic data for the mean relative fluorescent intensity (MRFI) calculated by MetaMorph software. The experiment was repeated five times, and data were represented as the mean ± standard deviation (*n* = 5). The scale bar represented 20 *μ*m. ^∗^Significant difference (*p* < 0.05, versus TP).

Ca^2+^ is known as a second messenger in organisms, of which the disequilibrium is believed to be involved in the pathogenesis of AD. A*β* can disrupt the calcium channels in cellular membrane, enhancing Ca^2+^ influx and then leading to the disequilibrium of calcium. Subsequently, the overload of intracellular Ca^2+^ is able to induce cytotoxicity and eventual cell death [30]. To further prove the neuroprotection of MC-PLGA-TP-NP, its effect on calcium influx of differentiated PC12 cells after the treatment of A*β*_1-42_ was evaluated. The resulting fluorescence images were shown in Figure 5, among which the green fluorescence corresponded to calcium deposition stained by Fluo-3-AM fluorescence and the red ones represented the lysosomes stained by LysoTracker Red in the PC12 cells, and the blue ones indicated the nuclei stained by DAPI (Figure 5(a)). It was evident that the coincubation of A*β*_1-42_ with the differentiated PC12 cells enhanced the calcium influx, and the mean relative fluorescent intensity (MRFI) of Fluo-3-AM fluorescence in the cells was approximately 3.6-fold higher than that in normal PC12cells (18.6 ± 3.68 for cells treated with A*β*_1-42_ only vs. 5.17 ± 1.24 for normal cells) as shown in Figure 5(b). The increase in intracellular Ca^2+^ levels was remarkably inhibited by the treatment of TP or MC-PLGA-TP-NP (Figures 5(a) and 5(b)). Furthermore, MC-PLGA-TP-NP exhibited the better inhibition effect on calcium influx (8.25 ± 2.04 vs. 12.62 ± 2.56 for TP, *p* < 0.05). In comparison of other groups, the strongest red fluorescence was observed, corresponding to the endocytosis of MC-PLGA-TP-NP into the cells. Colocalization of both the red and the green stains showing orange-yellow color indicated that plenty of MC-PLGA-TP-NP was transported into lysosome where lysosomal acidification drove Ca^2+^ influx [31]. In combination, the obtained data suggested that MC-PLGA-TP-NP may exert a better neuroprotective activity in the AD cellular model via following pharmacological mechanisms at least [1] inhibiting cytotoxicity and increasing the intracellular Ca^2+^ influx induced by A*β*, [2] attenuating the oxidative stress in cells. However, whether MC-PLGA-TP-NP has an obvious therapeutic effect on AD models in vivo has not been studied. We are aware that our claims would require confirmation after in vivo animal tests, including biodistribution and therapy efficiency of MC-PLGA-TP-NP on the transgenic animal model of AD.

## 4. Conclusion

We successfully prepared MC-PLGA-TP-NP showing sustained-release profile and better transcellular permeability to Caco-2 cells in comparison of TP. In addition, MC-PLGA-TP-NP significantly reduced the cytotoxicity, attenuated the oxidative stress, and inhibited the increase of the intracellular Ca^2+^ influx in differentiated PC12 cells induced by A*β*_1-42_. Therefore, it can be concluded that MC-PLGA-TP-NP should be a more promising preparation of TP, which may exert a better neuroprotective activity in the AD cellular model.

## Data Availability

The data used to support the findings of this study are available from the corresponding author upon request.
